# Sexual transmission of hepatitis E virus via vaginal and rectal routes in a rabbit model

**DOI:** 10.1099/jgv.0.002231

**Published:** 2026-04-01

**Authors:** Manyu Li, Wenjun Wan, Zeyu Song, Wenwen Sun, Qiyu He, Haiwei Zhou

**Affiliations:** 1 Division I of In Vitro Diagnostics for Infectious Diseases, Institute for In Vitro Diagnostics Control, National Institutes for Food and Drug Control, Beijing, PR China; 2 State Key Laboratory of Drug Regulatory Science, Beijing, PR China; 3 Institute of Human Virology, Department of Pathogen Biology and Biosecurity, Key Laboratory of Tropical Disease Control of Ministry of Education, Zhongshan School of Medicine, Sun Yat-sen University, Guangzhou, PR China

**Keywords:** adverse pregnancy outcomes, animal models, hepatitis E virus, pregnancy, sexual transmission

## Abstract

Hepatitis E virus (HEV) is a major cause of acute hepatitis worldwide. While faecal–oral transmission is well-established, the potential for sexual transmission remains unclear. This study aimed to investigate whether HEV can be transmitted through sexual routes using a rabbit model. A total of 80 rabbits were inoculated intravaginally or intrarectally with HEV, compared to intravenous challenge. To assess hormonal influence, rabbits were pre-treated with medroxyprogesterone acetate (MPA) or pregnant mare serum gonadotrophin (PMSG) prior to vaginal inoculation. Also, 12 pregnant rabbits were injected with HEV by vaginal and intravenous routes, respectively, to evaluate adverse outcomes. Viral shedding, seroconversion, viral loads in tissues, histopathology and vaginal microbiota were analysed. Our results showed that HEV can be transmitted through both vaginal and rectal routes, establishing productive but less efficient infections than intravenous inoculation. Crucially, the MPA dramatically enhanced vaginal susceptibility, leading to prolonged viral shedding, higher systemic viral loads and 100% seroconversion, compared to partial infection in PMSG-treated and untreated controls. Vaginal infection in pregnant rabbits resulted in significant adverse outcomes, including abortion (66.7%) and vertical transmission. Furthermore, vaginal inoculation altered the local microbiota and transcriptome, suggesting a remodelled genital tract microenvironment. Our study provided the first direct experimental evidence that HEV can be sexually transmitted via vaginal and rectal routes. The progesterone-mediated enhancement of susceptibility and the severe pregnancy outcomes highlighted its public health risk, particularly for women.

## Data Summary

All data are presented in the main figures. Raw sequencing data, microscopy images, materials and sequence information are available upon request. The sequence data are available from the NCBI nucleotide database (https://www.ncbi.nlm.nih.gov/nucleotide/), and the GenBank accession number is PX905820.

## Introduction

Hepatitis E virus (HEV) is the major cause of acute viral hepatitis globally, causing an estimated 20.1 million infections and 3.3 million symptomatic cases per year [1]. Among the eight main genotypes that have been identified, HEV genotype 1 (HEV1) to HEV genotype 4 (HEV4) mainly cause human infections [2]. HEV1 and HEV genotype 2 (HEV2) only infect humans, which can cause large outbreaks and epidemics in developing regions. HEV genotype 3 (HEV3) and HEV4 are dominant in developed regions and mainly lead to sporadic cases. HEV infection is usually self-limiting in general individuals, but it can lead to chronic infections in immunocompromised individuals [3]. In pregnant women, HEV infection can lead to high mortality rates and adverse pregnancy outcomes [4].

The main transmission routes of HEV include faecal–oral route, zoonotic transmission, blood transfusion and vertical transmission [1]. HEV1 and HEV2 are usually transmitted by faecal–oral route [3]. HEV3 and HEV4 mainly spread by zoonotic transmission, which is caused by consuming contaminated food or by contacting infected animals [3]. Recent evidence highlights another possible route of transmission: through sexual contact. HEV RNA has been detected in the semen of infected men, which continued for over 9 months after the end of viraemia [5,6]. Also, Cong *et al*. found that HEV RNA and/or antigens were detected in the vaginal secretions of women, and HEV can replicate in the female genital tract in animal models [7]. These results indicate the possibility of sexual transmission of HEV.

Many viruses, including HEV, human immunodeficiency virus (HIV) and Zika virus (ZIKV), can cause adverse outcomes in pregnant women [4,8,12]. Sexual transmission of ZIKV during pregnancy can lead to a high maternal mortality rate, abortion and foetal brain infection [13,14]. Pregnant women tend to have severe consequences when infected with HEV, including high mortality rate, stillbirth and abortion [4]. The possibility of HEV transmission through sexual contact, including vaginal and anal intercourse, still needs further investigation. Thus, the rabbit model is suitable for this study.

The vaginal microbiota has been shown to be related to the transmission and disease progression of multiple viruses, including HIV and human papillomaviruses [15,16]. The alteration of vaginal microbiota may be related to immune responses to inflammation, promoting the transmission of viruses [16]. So far, the relationship between HEV infection through vaginal and vaginal microbiota has not been studied yet.

The rabbit model has been used in studies of HEV, which can stimulate the HEV infection signs [17,19]. In this study, we investigated different transmission routes of HEV in a rabbit model. We also explored the vaginal microbiota changes in rabbits infected with HEV by vaginal transmission. Furthermore, we examined the HEV replication and adverse outcomes following vaginal infection of pregnant rabbits with HEV and its impact on fetuses.

## Methods

### Animals

Eighty-eight 3-month-old Japanese white rabbits (58 female and 30 male, weighing about 2–3 kg) were obtained from the Department of Laboratory Animal Science of National Institutes for Food and Drug Control. Also, twelve 7-month-old female Japanese white rabbits (weighing about 5–6 kg) were randomly selected from the Department of Laboratory Animal Science of National Institutes for Food and Drug Control. All animals were housed in individual cages with food and water available *ad libitum*. To establish the baseline for all parameters, serum and faeces samples from these rabbits were taken once per week for two consecutive weeks. To confirm that no specimens were positive for HEV RNA or antibodies against HEV, enzyme-linked immunosorbent assay (ELISA) and reverse transcription-nested PCR were conducted. The animal experiments were approved by the Committee of Laboratory Animal Welfare and Ethics of National Institutes for Food and Drug Control (2022B012).

### Viruses

The HEV strain (China, 2012, GenBank: JQ768461) was isolated from rabbit faeces and propagated *in vivo* in Japanese white rabbits. To confirm the sequence of the propagated virus, the ORF 2 was amplified by PCR and subjected to Sanger sequencing. The obtained sequence (PX905820) was identical to the original GenBank reference sequence (JQ768461). To prepare the inocula, the faeces were diluted in sterile PBS to make 10% (wt/vol) suspensions, and the suspensions were then filtered through 0.45 and 0.22 µm filters. The inocula, with a concentration of 5.0×10^6^ copies per millilitre as determined by reverse transcription quantitative real-time polymerase chain reaction (RT-qPCR) [20], were stored aliquoted at −80 °C.

### Inoculation of animals

Ninety rabbits (50 female and 40 male) were randomly divided into four groups: Vaginal, Rectal, Oral, Intravenous and Mock. Group Vaginal included ten female rabbits, and groups Rectal, Oral, Intravenous and Mock included ten female rabbits and ten male rabbits, respectively. We have previously measured the infectious HEV particles by challenging rabbits with different dilutions of HEV-3ra inoculum [21]. We established the 50% rabbit infectious dose (RID₅₀) – the dose at which half of the inoculated rabbits become infected – to be 2.5×10³ RNA copies as measured by our current RT-qPCR methods. Our previous studies [17,18] indicated that an inoculum of ~10³ RID₅₀ is optimal for investigating HEV infection and pathogenesis. Thus, in this study, each rabbit was administered 5×10⁶ copies of HEV-3ra inoculum, which was equivalent to 2×10^3^ RID₅₀. Group Vaginal were vaginally inoculated with 1 ml of HEV inoculum which had a viral load of 5.0×10^6^ copies per millilitre, resulting in a total dose of 5.0×10^6^ copies of HEV per animal. Similarly, groups Rectal, Oral and Intravenous were rectally, orally and intravenously inoculated with 5.0×10^6^ copies of HEV per animal, respectively. Rabbits in group Mock were inoculated intravenously with a rabbit faeces suspension without HEV infection as the negative control. The inoculation dose was chosen by previous studies which successfully established reproducible HEV infection and observed typical virological and pathological changes [17,18].

Eighteen female rabbits were randomly assigned into three groups: medroxyprogesterone acetate (MPA), pregnant mare serum gonadotrophin (PMSG) and negative control (NC). Group MPA received a subcutaneous injection of 5 mg of MPA (Macklin, Shanghai, China) twice a week for 1 week prior to virus inoculation [22]. Group PMSG was injected intramuscularly with 100 IU of PMSG (Solarbio, Beijing, China) before virus inoculation [23]. Then, groups MPA and PMSG were vaginally inoculated with 5.0×10^6^ copies of HEV. Group NC received a rabbit faeces suspension without HEV infection vaginally as the negative control.

Twelve 7-month-old female rabbits were randomly assigned into two groups (PI and PV), each with six rabbits. All rabbits (*n*=12) successfully mated with healthy male rabbits (gestational day 0). After their pregnancy was confirmed, rabbits in group PI and PV were inoculated with 5.0×10^6^ copies of HEV intravenously and vaginally, respectively.

### Sample collection

Serum samples and faecal samples of animals were obtained on the day of inoculation (day 0) and then were collected consecutively. Vaginal washes were taken 1–7 days post-infection (dpi), 14 dpi, 21 dpi and 28 dpi. At 4 and 8 weeks post-inoculation (wpi), three rabbits of group Vaginal, six rabbits of group Rectal (three female, three male), six rabbits of group Intravenous (three female, three male) and six rabbits of group Mock (three female, three male) were euthanized, respectively. All the remaining rabbits were monitored for at least 8 weeks. The remaining animals were euthanized at the end of the study. The tissues were rapidly collected after the animal was rapidly euthanized. For each tissue, the samples were both stored at −80 °C and fixed with 4% paraformaldehyde.

### Extraction of HEV RNA and RT-qPCR

HEV RNA was extracted by the EasyPure^®^ Viral DNA/RNA Kit (TransGen Biotech, China) following the manufacturer’s instructions. All RNA samples were eluted with 20 µl of distilled water and then stored at −80 °C until analysis. Then, the HEV RNA was quantitated by one-step quantitative real-time reverse transcription PCR using GoTaq^®^ qPCR Master Mix (Promega, USA) on an ABI Prism 7500 Sequence Detection System (Thermo Fisher Scientific, USA). The primers and probe used in the RT-qPCR were designed by Jothikumar *et al*. [20].

### HEV antibody and antigen detection

Total anti-HEV antibodies in rabbit serum samples and HEV antigen in rabbit serum samples and cells were tested by ELISA using the commercial kit (Wantai, Beijing, China) according to the manufacturer’s instructions. The assay was read at 450 nm for HEV antibody and antigen, and when the S/CO value >1, it was considered to be positive.

### Measurement of alanine aminotransferase, aspartate aminotransferase, total bilirubin and progesterone levels

The rabbit serum samples were tested for alanine aminotransferase (ALT), aspartate aminotransferase (AST) and total bilirubin (TBil) by Chemray 240 (Rayto, Shenzhen, China). When the ALT, AST or TBil levels increased by more than two times the baseline value before infection, the rabbit was considered to have liver inflammation. The progesterone levels of rabbit serum samples were tested by ELISA using the commercial kit (Jianglai, Shanghai, China) according to the manufacturer’s instructions.

### Histopathology

Rabbit tissues, including liver, intestine and vagina, were dissected and fixed in formalin. Then, tissue sections were stained with haematoxylin and eosin (H and E) staining and subjected to image acquisition using PANNORAMIC DESK (3DHISTECH, Budapest, Hungary).

### RNA sequencing analysis

The rabbit tissue samples were flash-frozen in liquid nitrogen and subjected to RNA sequencing (Novogene, Beijing, China). The libraries were sequenced on an Illumina NovaSeq platform, and 150 bp paired-end reads were generated. Differential expression analysis of two groups was performed using the DESeq2R package (1.20.0). Cutoff criteria (*P*-value <0.05 and |logFC|≥1.0) were applied to identify significantly differentially expressed genes (DEGs). The clusterProfiler R package was used to conduct the Gene Ontology (GO) enrichment analysis of DEGs. GO terms with corrected *P*-value less than 0.05 were considered significantly enriched by DEGs. Kyoto Encyclopedia of Genes and Genomes (KEGG) pathway enrichment analysis of DEGs was analysed by the clusterProfiler R package.

### 16S rRNA sequencing of the vaginal microbiota

The bacterial DNA of rabbits’ vaginal swab samples was extracted at Novogene Bioinformatics Technology Co., Ltd., using Tiangen kits (Tiangen, Beijing, China) according to the manufacturer’s instructions. The V3 and V4 regions of the 16S rRNA gene were amplified by PCR. All samples were paired-end sequenced on the Illumina HiSeq X Ten platform (insert size 350 bp, read length 150 bp) at Novogene Bioinformatics Technology Co., Ltd. Quality filtering on the raw tags was performed using the fastp (Version 0 .23 . 1) software to obtain high-quality Clean Tags. For the Effective Tags obtained previously, denoise was performed with DADA2 or deblur module in the QIIME2 software (Version QIIME2-202202). In order to analyse the diversity, richness and uniformity of the communities in the sample, alpha diversity was calculated in QIIME2. In order to evaluate the richness of microbial community and sample size, species accumulation boxplot can be used to visualize, which is performed with the vegan package in R software. In order to evaluate the complexity of the community composition and compare the differences between samples (groups), beta diversity was calculated based on weighted and unweighted UniFrac distances in QIIME2.

### Statistical analysis

Statistical analysis was performed using the SPSS PASW Statistics v18.0 statistical software package (SPSS, Inc., USA, http://www.ibm.com/cn/). Data were compared using Student’s t-test, Mann-Whitney test and one-way ANOVA test. A correction for multiple comparisons was applied using the Bonferroni method. Adjusted *P*-values were reported throughout the manuscript. A *P*-value of <0.05 was considered significant.

## Results

### HEV can be transmitted vaginally in rabbits

**Fig. 1.**
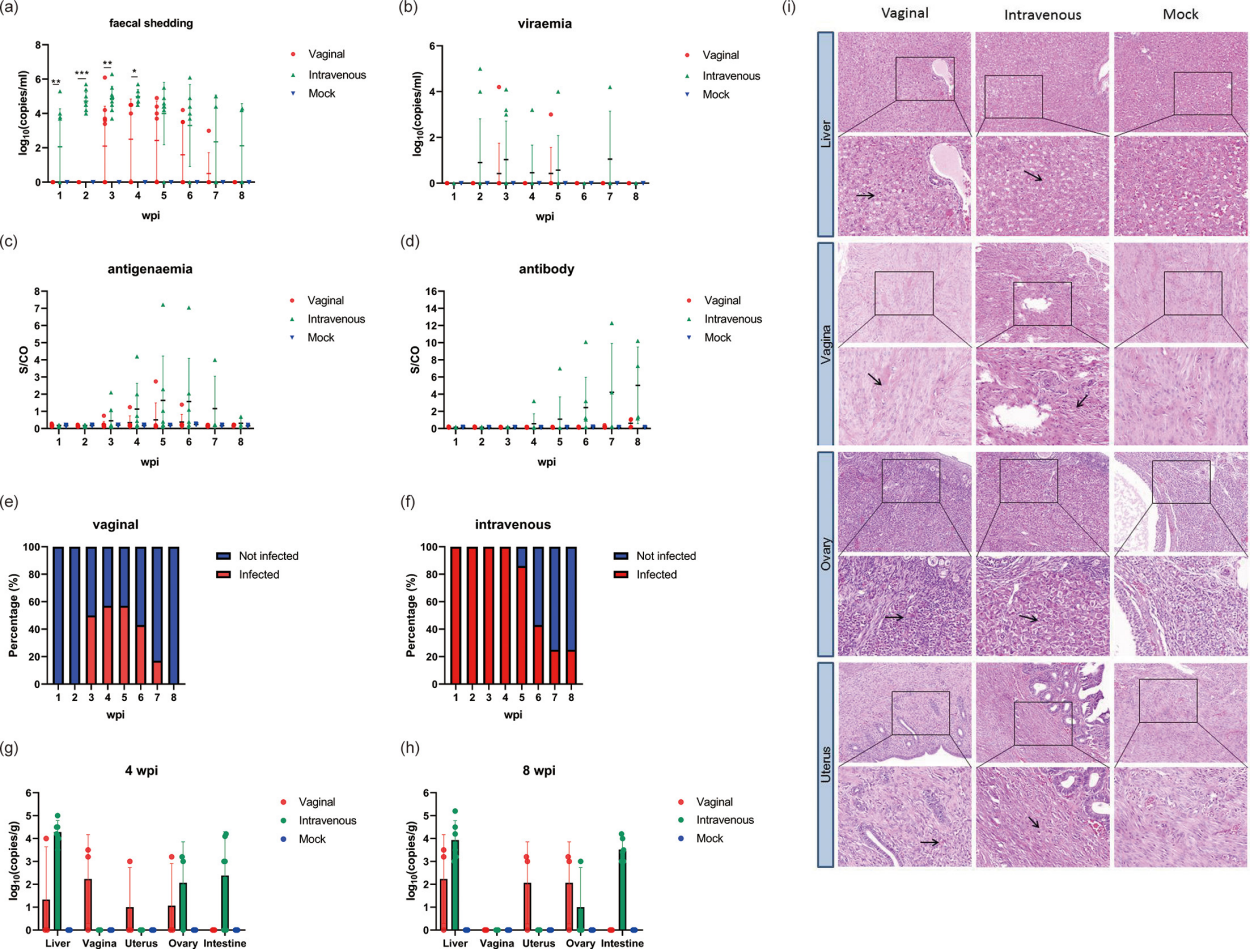
Clinical manifestations in rabbits infected with HEV via different transmission routes. (**a–d**) Dynamics of HEV infection. Faecal viral shedding (**a**), viraemia (**b**), antigenaemia (**c**) and antibody seroconversion (**d**) were observed in rabbits following HEV inoculation via vaginal and intravenous routes. Data were presented as scatter plots where each symbol represents one animal. The vertical bars represented the sd, and the horizontal bars indicated the group median. Red symbols represented group Vaginal, green symbols represented group Intravenous, and blue symbols represented group Mock. (**e, f**) Durations of faecal viral shedding in all groups. (**g, h**) HEV RNA loads in the liver, vagina, uterus, ovary and intestine in all groups at 4 and 8 wpi. Data were presented as scatter plots where each symbol represents one animal. The vertical bars represented the sd, and the horizontal bars indicated the group median. Red symbols represented group Vaginal, green symbols represented group Intravenous, and blue symbols represented group Mock. (**i**) Representative H and E staining of the liver, vagina, ovary and uterus sections in all groups. Focal hepatocellular necrosis and inflammatory cell infiltration in the liver tissues of both the Vaginal and Intravenous groups (**i**). Haemorrhage and structural disruption were observed in the vagina, ovary and uterus tissues of both the Vaginal and Intravenous groups. Black arrows indicate areas of pathological changes. *P<0.05, **P<0.01 and ***P<0.001.

It remains unknown whether HEV can be transmitted through sexual transmission vaginally. To address this issue, we established a rabbit model of HEV sexual transmission. Rabbits in group Vaginal and Intravenous were inoculated with 5.0×10^6^ copies of HEV intravaginally and intravenously, respectively. Compared to intravenous inoculation, intravaginal challenge with HEV led to a successful but less efficient infection, as evidenced by delayed and shortened faecal viral shedding and a lower seroconversion rate (Fig. 1).

A total of 50 and 100% of rabbits in Vaginal and Intravenous groups showed faecal virus shedding, respectively. Specifically, viral RNA was detected in faeces as early as 1 wpi in the majority of rabbits of the Intravenous group (Fig. 1a). However, in the Vaginal group, faecal virus shedding initiated at 3 wpi in the majority of rabbits and became undetectable levels by 7 wpi (Fig. 1a). Consequently, the mean duration of faecal virus shedding was significantly shorter in the Vaginal group (3.5±0.7 weeks) compared to the Intravenous group (5.1±1.2 weeks) (Fig. 1e, f).

Viraemia was observed in all groups since 2 wpi (Fig. 1b), and antigenaemia was detected in all groups from 3 wpi (Fig. 1c). While all intravenous-inoculated rabbits seroconverted by 4 wpi, only 50% of the vaginal-inoculated rabbits developed anti-HEV antibodies (Fig. 1d).

At 4 and 8 wpi, we collected liver, vagina, uterus and ovary tissues from rabbits in all groups to measure HEV RNA (Fig. 1g, h). The results indicated that intravenous injection of HEV into rabbits resulted in higher levels of viral RNA found in liver at 4 and 8 wpi than in other groups. Vaginal inoculation of HEV resulted in higher levels of HEV RNA detected in the vagina tissues at 4 wpi and undetectable levels by 8 wpi, demonstrating localized infection following vaginal exposure. Consistent with viral replication, the histopathological analysis revealed focal hepatocellular necrosis and inflammatory cell infiltration in the liver tissues of both the Vaginal and Intravenous groups (Fig. 1i). Haemorrhage and structural disruption were observed in the vagina, ovary and uterus tissues of both the Vaginal and Intravenous groups. No obvious pathological changes were observed in the Mock group.

Together, our results showed that HEV can be transmitted by vaginal inoculation in rabbits, resulting in both systemic infection and local genital tract pathology.

### MPA and PMSG treatment affect HEV vaginal transmission

In vaginal viral infection models of other viruses, different phases of the oestrus cycle may affect the host susceptible to infection. Given that only 50% of rabbits in group Vaginal showed HEV infection signs, we hypothesized that hormone treatment can affect HEV vaginal transmission susceptibility. To test this, we treated rabbits with MPA or PMSG prior to vaginal HEV challenge.

**Fig. 2.**
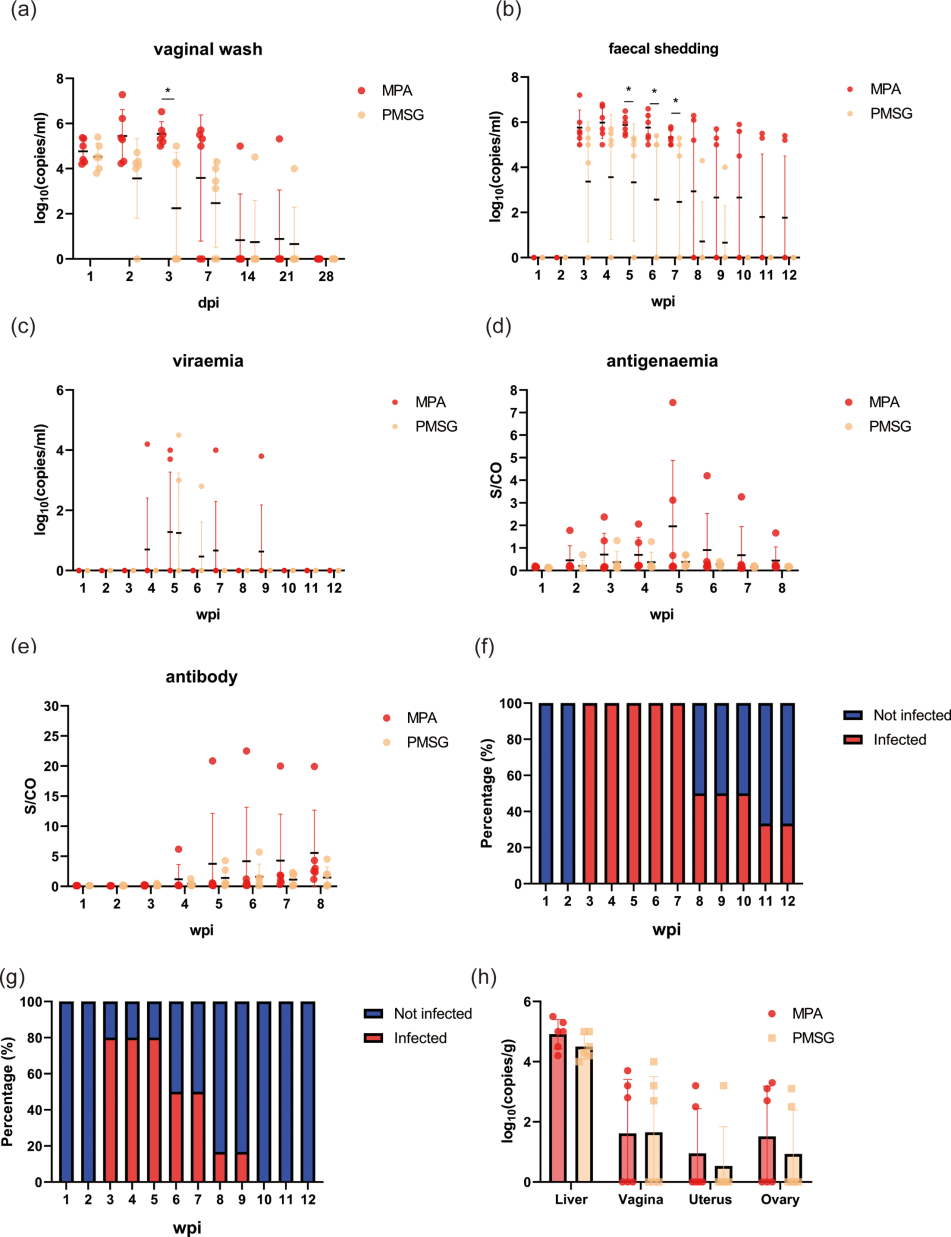
HEV infection in rabbits via vaginal route following MPA and PMSG treatment. (**a**) HEV RNA detection in vaginal wash samples. Data were presented as scatter plots where each symbol represents one animal. The vertical bars represented the sd, and the horizontal bars indicated the group median. Red symbols represented group MPA, and orange symbols represented group PMSG. (**b–e**) Faecal viral shedding, viraemia, antigenaemia and antibody seroconversion in infected rabbits. Data were presented as scatter plots where each symbol represents one animal. The vertical bars represented the sd, and the horizontal bars indicated the group median. Red symbols represented group MPA, and orange symbols represented group PMSG. (**f**) Duration of faecal viral shedding in the PMSG group. (**g**) Duration of faecal viral shedding in the MPA group. (**h**) HEV RNA loads in the liver, vagina, uterus and ovary in all groups. Data were presented as scatter plots where each symbol represents one animal. The vertical bars represented the sd, and the horizontal bars indicated the group median. Red symbols represented group MPA, and orange symbols represented group PMSG. *P<0.05.

We found that MPA treatment dramatically enhanced HEV infection compared to PMSG treatment. After inoculation, the viral load in vaginal wash was measured at 1–7 dpi, 14 dpi, 21 dpi and 28 dpi. Viral shedding in vaginal wash was more persistent in the MPA group. HEV RNA was detected in the vaginal wash of 100% (6 out of 6) of rabbits in group MPA from 1 to 3 dpi, 66.7% (4 out of 6) on 7 dpi and 16.7% (1 out of 6) on 14 and 21 dpi (Fig. 2a). In group PMSG, HEV RNA was detected in the vaginal wash of 100% (6 out of 6) of rabbits on 1 dpi, 83.3% (5 out of 6) on 2 dpi, 50.0% (3 out of 6) on 3 dpi, 66.7% (4 out of 6) on 7 dpi and 16.7% (1 out of 6) on 14 and 21 dpi (Fig. 2a). On 28 dpi, no HEV RNA was detected in the vaginal wash of all the rabbits.

MPA treatment led to a prolonged faecal virus shedding (mean duration: 7.2±2.5 weeks vs. 3.3±2.9 weeks in the PMSG group) (Fig. 2b). By detecting the viral RNA in faeces and serum, we found that faecal virus shedding was observed until 3 wpi, and viraemia was observed until 4 wpi (Fig. 2c). The viral loads of group MPA were significantly higher than those of group PMSG from 3 to 12 wpi. The S/CO levels of HEV antigen in group MPA were also higher than those in group PMSG (Fig. 2d). Until the end of observation, 100% (6 out of 6) of rabbits in group MPA showed seroconversion of anti-HEV antibodies, and only 50.0% (3 out of 6) of group PMSG were positive for anti-HEV antibodies (Fig. 2e). The mean faecal virus shedding duration of groups MPA and PMSG was 9.7±2.0 and 5.8±3.3 weeks, respectively (Fig. 2f, g).

MPA-treated animals also exhibited more severe hepatic dysfunction and higher viral loads in reproductive tissues. The ALT, AST and TBil levels were elevated compared to non-hormone-treated infected rabbits since 3 wpi (Fig. S1A–S1C, available in the online Supplementary Material). At 8 wpi, the liver, vagina, uterus and ovary tissues were collected and were measured for HEV RNA (Fig. 2h). The results showed that HEV RNA was detected in livers of all rabbits, with a mean viral load of 4.9±0.5 log_10_ copies per millilitre and 4.5±0.4 log_10_ copies per millilitre in groups MPA and PMSG, respectively. HEV RNA was detected in 50.0% (3 out of 6) of vagina tissues of rabbits in groups MPA and PMSG, with a mean viral load of 3.2±0.5 log_10_ copies per millilitre and 3.3±0.7 log_10_ copies per millilitre, respectively. A total of 50.0% (3 out of 6) of uterus tissues and ovary tissues of group MPA were found to be positive for HEV RNA. In group PMSG, 16.7% (1 out of 6) of uterus tissues and 33.3% (2 out of 6) of ovary tissues were positive for HEV RNA. These results demonstrated that a progesterone-dominant state significantly enhances vaginal susceptibility to HEV infection, suggesting a hormonal basis for transmission risk.

### Vaginal HEV infection of dams resulted in adverse pregnancy outcomes

**Fig. 3.**
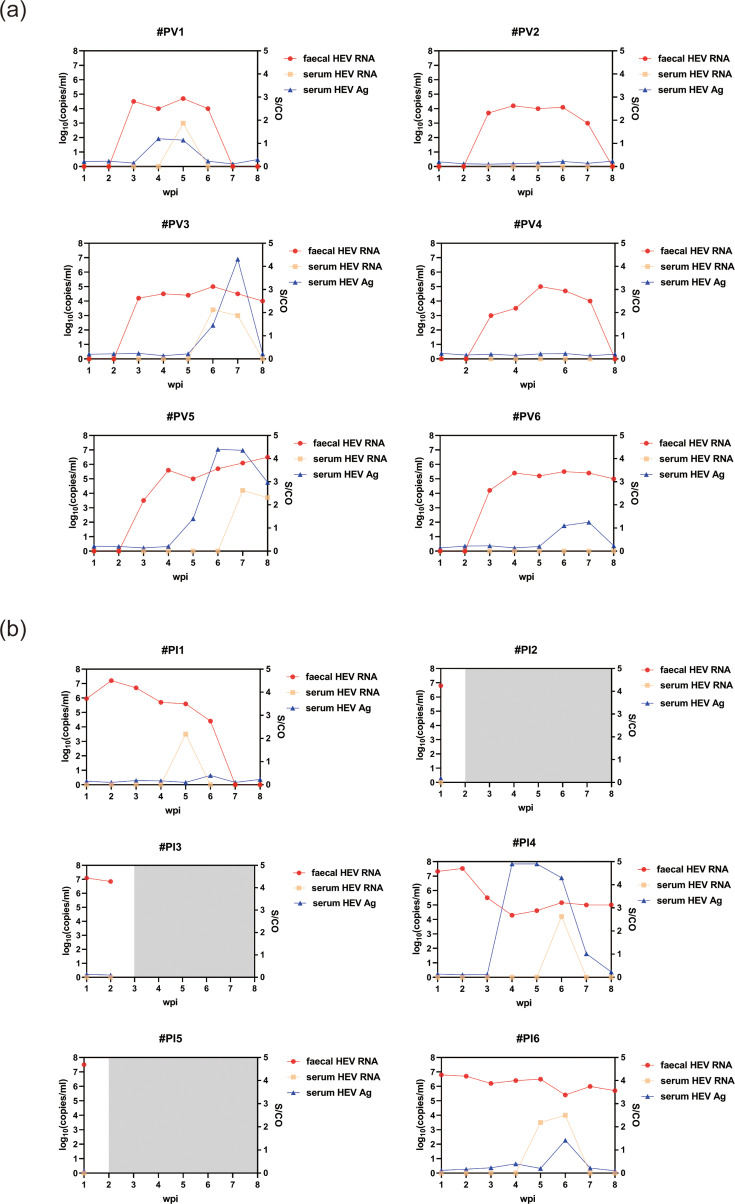
HEV infection signs of pregnant rabbits after HEV vaginal and intravenous inoculation. (**a**) Dams in group PV, inoculated with HEV vaginally, showed HEV infection signs including faecal viral shedding, viraemia and antigenaemia. (**b**) Dams in group PI, which were inoculated with HEV intravenously, showed HEV infection signs including faecal viral shedding, viraemia and antigenaemia.

Since HEV infection during pregnancy can lead to severe consequences, we hypothesized that HEV infection by vaginal transmission during pregnancy may cause adverse outcomes. To test this hypothesis, we infected 12 pregnant rabbits in different ways: group PI (*n*=6) was inoculated with 5.0×10^6^ copies of HEV intravenously, and group PV (*n*=6) was inoculated with 5.0×10^6^ copies of HEV vaginally. All rabbits in both groups showed faecal virus shedding, with PI starting faecal virus shedding from 1 wpi and PV from 3 wpi (Fig. 3a, b). All alive pregnant rabbits (100%, 3 out of 3) in group PI showed viraemia and antigenaemia, and 50.0% (3 out of 6) of group PV showed viraemia and antigenaemia (Fig. 3a, b). Their progesterone levels were not significantly different (Fig. S2).

**Fig. 4.**
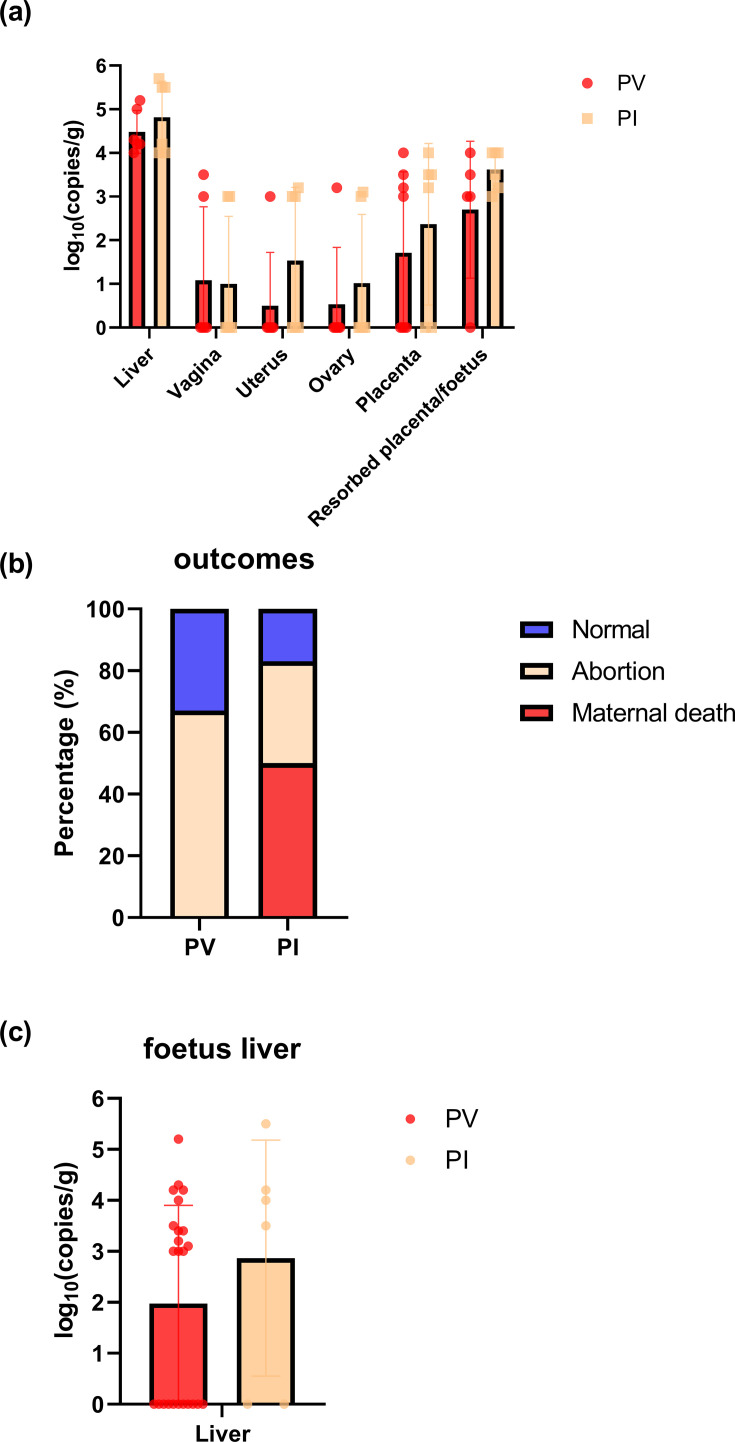
HEV-related adverse pregnancy outcomes of pregnant rabbits following vaginal and intravenous HEV inoculation. (**a**) HEV RNA detection in various tissues from PV and PI groups. Data were presented as scatter plots where each symbol represents one animal. The vertical bars represented the sd, and the horizontal bars indicated the group median. Red symbols represented group PV, and orange symbols represented group PI. (**b**) Adverse pregnancy outcomes in PV and PI groups following HEV inoculation. (**c**) HEV RNA detection in foetal livers from PV and PI groups. Data were presented as scatter plots where each symbol represents one animal. The vertical bars represented the sd, and the horizontal bars indicated the group median. Red symbols represented group PV, and orange symbols represented group PI.

Then, we examined the HEV infection of the maternal and foetal tissues (Fig. 4a). We detected HEV RNA in the liver, vagina, uterus, ovary, placenta and resorbed placenta/fetus tissues in both groups. The viral loads in these tissues did not show significant differences between the two groups (*P*>0.05).

Both intravenous and vaginal infection of pregnant rabbits resulted in severe adverse outcomes, including abortion and maternal death. We observed that 50.0% (3 out of 6) of group PI showed maternal death, 33.3% (2 out of 6) showed abortion and 16.7% (1 out of 6) had normal delivery (Fig. 4b). In group PV, 66.7% (4 out of 6) showed abortion, and 33.3% (2 out of 6) had normal delivery (Fig. 4b).

We also detected the HEV infection of foetus liver tissues (Fig. 4c). In group PV, 54.2% (13 out of 24) of foetus liver tissues were positive for HEV RNA. In group PI, HEV RNA was detected in 66.7% (4 out of 6) of foetus liver tissue. Their viral loads did not have significant differences (*P*>0.05). These results provided evidence of vertical transmission following vaginal inoculation. Although the intravenous infection group exhibited higher maternal mortality, the vaginal route posed a significant threat to foetal survival, causing a high rate of abortion.

### HEV vaginal inoculation led to vaginal microbiota alteration

**Fig. 5.**
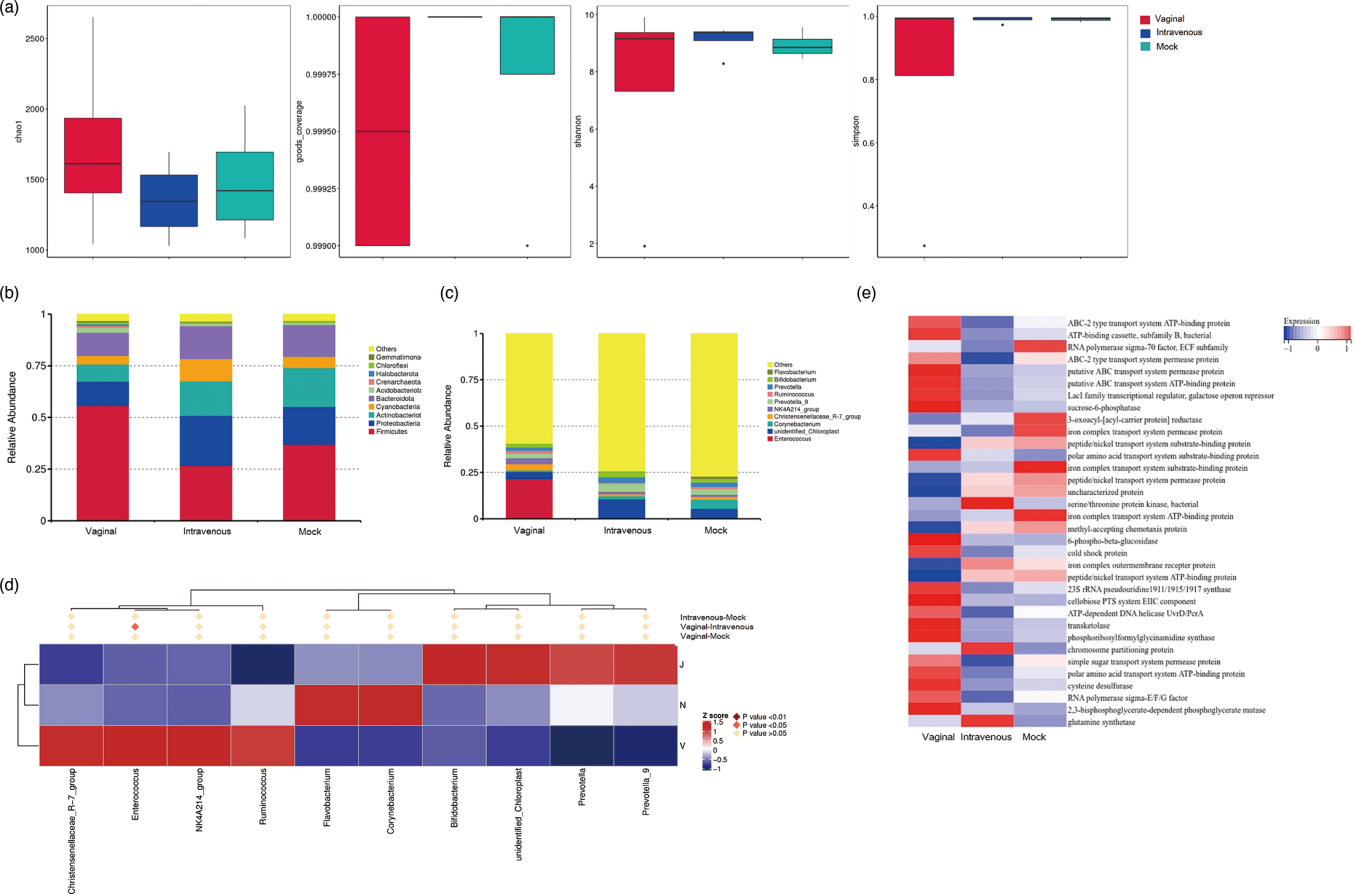
Characterization of the vaginal microbiota in the Vaginal, Intravenous and Mock groups. (**a**) Alpha diversity indices, including Chao1, Goods coverage, Shannon and Simpson, of the vaginal microbiota. (**b, c**) Relative abundances of the vaginal microbiota at the phylum and genus levels. (**d**) Differences in the relative abundances of the vaginal microbiota at the genus level among three groups by metagenomeSeq, and there were significant differences between group Vaginal and group Intravenous on the genus level of *Enterococcus*. (**e**) The PICRUSt2 prediction of functional profile analysis.

The vaginal microbiome of groups Vaginal, Intravenous and Mock was characterized using 16S rRNA gene sequencing. The abundance and evenness of microbiota in each group are shown. Alpha diversity indices, including Chao1, goods_coverage, Shannon and Simpson, were used to assess the ecological diversity within each group (Fig. 5a). Results showed that no significant difference was observed in the species richness or diversities among these groups.

Then, the taxonomic profiling of microbiota was investigated. The relative abundance at the phylum level showed that the rabbit vagina microbiome of these groups was dominated by *Firmicutes*, *Proteobacteria*, *Actinobacteriota*, *Bacteroidota* and *Cyanobacteria* on the phylum level (Fig. 5b). On the genus level, the average relative abundances of *Enterococcus*, *Christensenellaceae_R-7_group*, *NK4A214_group* and *Ruminococcus* of group Vaginal (21.5%, 3.3%, 3.3% and 1.7%) were higher than other groups (Fig. 5c).

By metagenomeSeq, we found that there were significant differences between group Vaginal and group Intravenous on the genus level of *Enterococcus* (Fig. 5d). By PICRUSt2 analysing, we found that the species in group Vagina were more involved in the ATP-binding system and the ABC transport system compared to other groups (Fig. 5e).

Also, by transcriptomic analysis of vaginal tissues from group Vaginal and group Mock, a total of 495 DEGs (Fig. S3A) were identified. The GO enrichment and KEGG analysis showed that most DEGs were enriched in different pathways (Fig. S3B and S3C). Genes related to the PI3K-AKT-mTOR signalling pathway were significantly expressed in the Vaginal group (Fig. S3D).

### HEV can also be transmitted by rectal route in rabbits

**Fig. 6.**
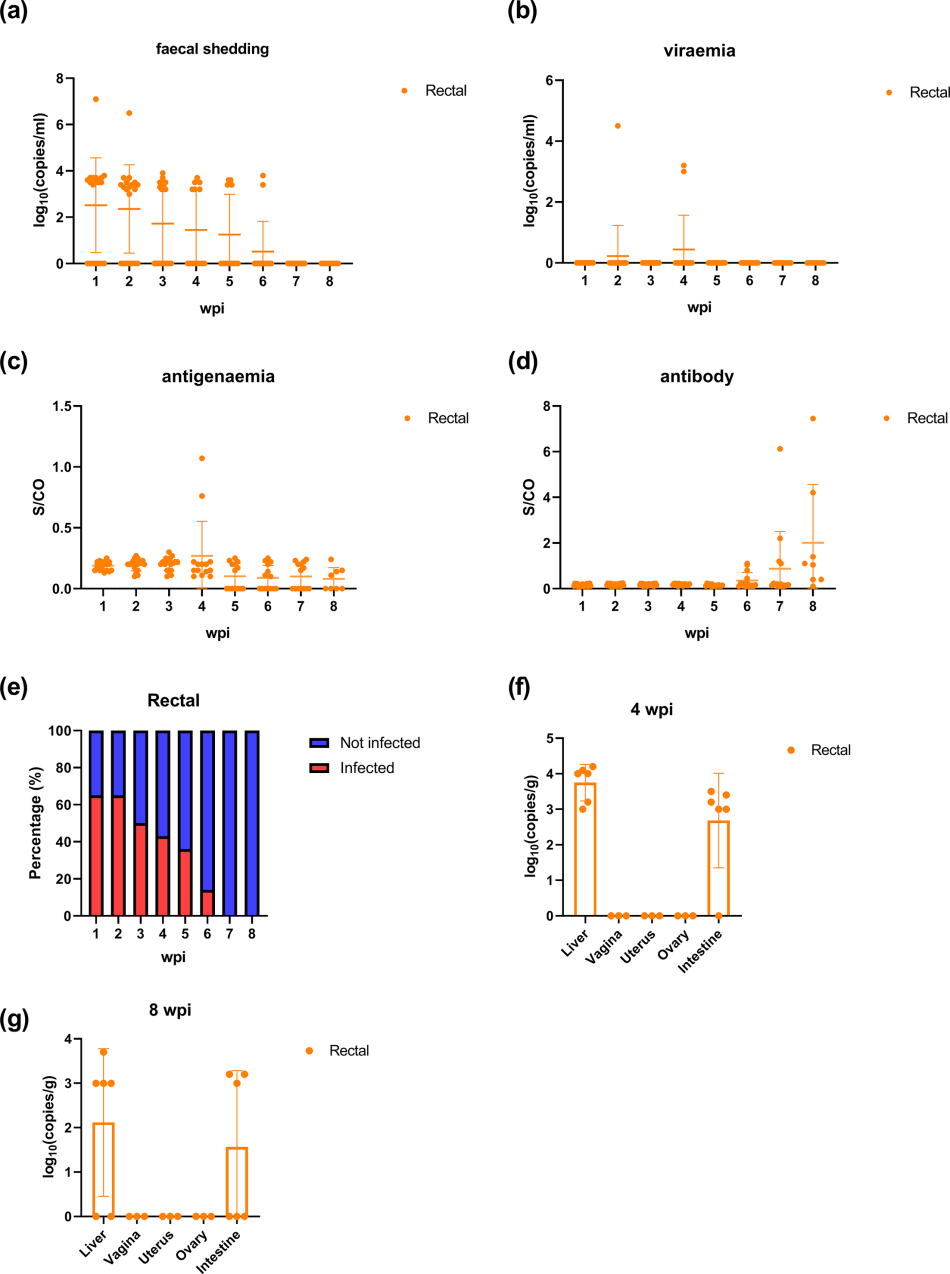
HEV infection in rabbits via rectal route. (**a–d**) Faecal viral shedding, viraemia, antigenaemia and antibody seroconversion in infected rabbits. Data were presented as scatter plots where each symbol represents one animal. The vertical bars represented the sd, and the horizontal bars indicated the group median. Orange symbols represented group Rectal. (**e**) Duration of faecal viral shedding of rabbits. (**f, g**) HEV RNA loads in the liver, vagina, uterus, ovary and intestine of rabbits at 4 wpi and 8 wpi. Data were presented as scatter plots where each symbol represents one animal. The vertical bars represented the sd, and the horizontal bars indicated the group median. Orange symbols represented group Rectal.

To explore other potential mucosal routes, we assessed rectal transmission. Twenty rabbits were inoculated with 5.0×10^6^ copies of HEV rectally. Fourteen out of twenty rabbits had faecal virus shedding since 1 wpi (Fig. 6a). Viraemia and antigenaemia were observed at 2 and 4 wpi (Fig. 6b, c). At the end of the study period (8 wpi), 70% of rabbits showed seroconversion for anti-HEV antibodies (Fig. 6d). Of the rabbits in the Rectal group, 65% exhibited faecal virus shedding, with an average duration of 3.1±1.7 weeks (Fig. 6e). At 4 and 8 wpi, we collected liver, vagina, uterus, ovary and intestine from rabbits to measure HEV RNA (Fig. 6f, g). Besides the liver, viral RNA was also detected in the intestine at 4 wpi, but its levels decreased by 8 wpi. No obvious pathological changes were observed in the intestine tissues of the Rectal group (Fig. S4).

As a comparison, we also conducted experiments on oral transmission. Twenty rabbits were inoculated with 5.0×10^6^ copies of HEV orally. However, HEV RNA was only detected in faecal samples from 2 rabbits (2 out of 20, 10.0%) at 1 wpi (Table S1). No viraemia or antigenaemia or seroconversion for anti-HEV antibodies was observed (Table S1).

## Discussion

HEV can be transmitted through several routes, including the faecal–oral route, zoonotic transmission, blood transfusions and vertical transmission [1,3]. In addition to the above routes, HEV has been detected in the ejaculate of infected men [5,6]. Recently, Cong *et al*. reported that infectious HEV is excreted into the vagina [7]. These results suggest that HEV may be transmitted sexually. However, whether HEV can be transmitted through the vagina or rectum remains poorly understood. In this study, we provided experimental evidence that HEV had the potential to be transmitted via the vaginal and rectal routes in a rabbit model. Vaginal transmission of HEV altered the vaginal microbiota, and different phases of the oestrous cycle may influence the rabbits' susceptibility to HEV vaginal inoculation. Moreover, vaginal inoculation of HEV can result in adverse pregnancy outcomes in pregnant rabbits.

In this study, we compared different mucosal pathways of HEV inoculation. We provided further evidence that HEV can be sexually transmitted, expanding our understanding of its transmission dynamics beyond traditional routes. The detection of HEV in semen [5,6] and its excretion into the vagina [7] suggested that sexual transmission may play a significant role in its spread, especially in certain high-risk populations. The total dose we administered via the vaginal and rectal routes in this study was 5.0×10^6^ copies, which fell entirely within the reported human ejaculate viral load range [5,6]. Our results showed that HEV RNA can be detected in vaginal washes, faeces and serum of rabbits inoculated via the vaginal and rectal routes. Although they exhibited milder signs than those inoculated intravenously, these results suggested potential transmission between males and females, as well as male-to-male transmission. Thus, these findings indicated the potential for HEV transmission via diverse sexual practices, including vaginal and anal intercourse, which could be particularly relevant for high-risk groups such as individuals with multiple sexual partners or those engaging in unprotected sex. Also, the differences in HEV susceptibility to different mucosal surfaces may indicate the potential molecular mechanisms underlying this tissue tropism. However, the possibility of these routes needs further investigation and validation by *in vitro* models of these distinct mucosal barriers in the future.

The alteration of the vaginal microbiota following HEV vaginal transmission was observed in our study. Changes in the vaginal microbiota can influence not only the host’s immune responses but also the susceptibility to other infections [24]. It has been reported that *Lactobacillus* was strongly associated with an increased risk to sexually transmitted infections, including HIV [25,26]. The vaginal microbiota plays a crucial role in immune defence and cytokine secretion, including IL-1β, IL-6, IL-8 and TNF-α [27]. Our data suggested that HEV infection may disrupt the vaginal microbiota balance. Also, we found the predominant enrichment of the ABC transporter within the vaginal microbiota after HEV inoculation by vaginal route. ABC transporters are linked to the adaptive immune system, and the ABC trafficking complex participates in antigen presentation by cytotoxic T cells [28].

Moreover, our results showed that hormones influence susceptibility to HEV vaginal inoculation. Hormones affect women with reproductive tracts at different stages of the menstrual cycle and after menopause [29]. The sex hormones can regulate the immune response [30], epithelial integrity [31] or the composition of the vaginal microbiota [32], all of which may impact the efficiency of viral transmission and persistence through the vagina. Progesterone is important in the maintenance of pregnancy [33]. It has been reported that progesterone can enhance HEV replication in cells [34]. And HEV-seropositive patients had a 30% progesterone receptor mutation, while HEV-seronegative patients only had a 14% mutation [35]. In this study, we showed that the viral loads and S/CO levels of HEV antigen of group MPA were significantly higher than those of group PMSG, indicating that hormones can influence the permissive and persistent natures of HEV infection in the female reproductive tract. Future studies should focus on whether these hormonal changes may affect HEV vaginal transmission in human beings.

HEV is known to cause severe outcomes during pregnancy, including abortion, stillbirth and maternal death [4]. Since progesterone is shown to be associated with HEV vaginal transmission in our study, we aimed to investigate whether vaginal inoculation with HEV could affect pregnancy outcomes. The adverse pregnancy outcomes were observed in pregnant rabbits following vaginal inoculation with HEV, and HEV RNA can be detected in placenta and foetal tissues. Our findings regarding the role of progesterone in enhancing HEV vaginal transmission indicated that hormonal changes during pregnancy could influence the susceptibility to HEV infection by vaginal route. Thus, it is crucial to investigate the mechanisms in the future, which may have significant implications for managing HEV in pregnant women.

In comparison, the oral administration of HEV in our study showed that HEV RNA was transiently detected in the faeces of 2 out of 20 animals at 1 wpi, but seroconversion or sustained viraemia was not observed in this group, indicating that no productive infection via the oral route was established in this study. Similarly, studies using different animal models have shown that oral inoculation resulted in low infection efficiency [36,40]. The reasons may be due to the lack of certain essential receptors in the animal intestines or the degradation of the infectious HEV particles in the intestinal tract [37,39].

There are several limitations in our study. First, the possibility of sexual transmission of HEV remains to be fully validated in clinical cases, and the underlying mechanisms need further investigations. Second, the infection experiments were conducted using a single viral inoculum dose. Future studies investigating the potential dose dependency of infection efficiency would be valuable. Third, the infectivity of HEV particles in semen or vaginal secretions in the rabbit model was not investigated in this study. Fourth, the intravenous route is not the main natural route of HEV transmission, and its scientific relevance and biological significance of experiments conducted under such artificial conditions still need to be further verified. These issues should be addressed in the future independent studies.

In conclusion, we used HEV3 and found that HEV had the potential to be transmitted via the vaginal and rectal routes in a rabbit model, which can affect vaginal microbiota. Hormonal treatment was related to the severity of HEV vaginal transmission. Also, vaginal inoculation of HEV could lead to adverse pregnancy outcomes. These results highlight the potential of HEV sexual transmission, which requires better prevention and control strategies for HEV, particularly in vulnerable populations such as pregnant women.

## Supplementary material

10.1099/jgv.0.002231 Uncited Supplementary Material 1.

## Abbreviations

ALT: alanine aminotransferase
AST: aspartate aminotransferase
DEGs: differentially expressed genes
dpi: days post-infection
ELISA: enzyme-linked immunosorbent assay
GO: Gene Ontology
H and E: haematoxylin and eosin
HEV1: HEV genotype 1
HEV2: HEV genotype 2
HEV3: HEV genotype 3
HEV4: HEV genotype 4
HEV: hepatitis E virus
HIV: human immunodeficiency virus
KEGG: Kyoto Encyclopedia of Genes and Genomes
MPA: medroxyprogesterone acetate
PMSG: pregnant mare serum gonadotrophin
RID₅₀: 50% rabbit infectious dose
wpi: weeks post-inoculation
ZIKV: Zika virus
