# Supplementary material for: Sexual transmission of hepatitis E virus via vaginal and rectal routes in a rabbit model
Source: J Gen Virol. 2026 Apr 1;107(4):002231. doi: 10.1099/jgv.0.002231 (PMC13043109; doi:10.1099/jgv.0.002231)
Supplement: Uncited Supplementary Material 1. [file jgv-107-02231-s001.pdf]

Supplementary Materials

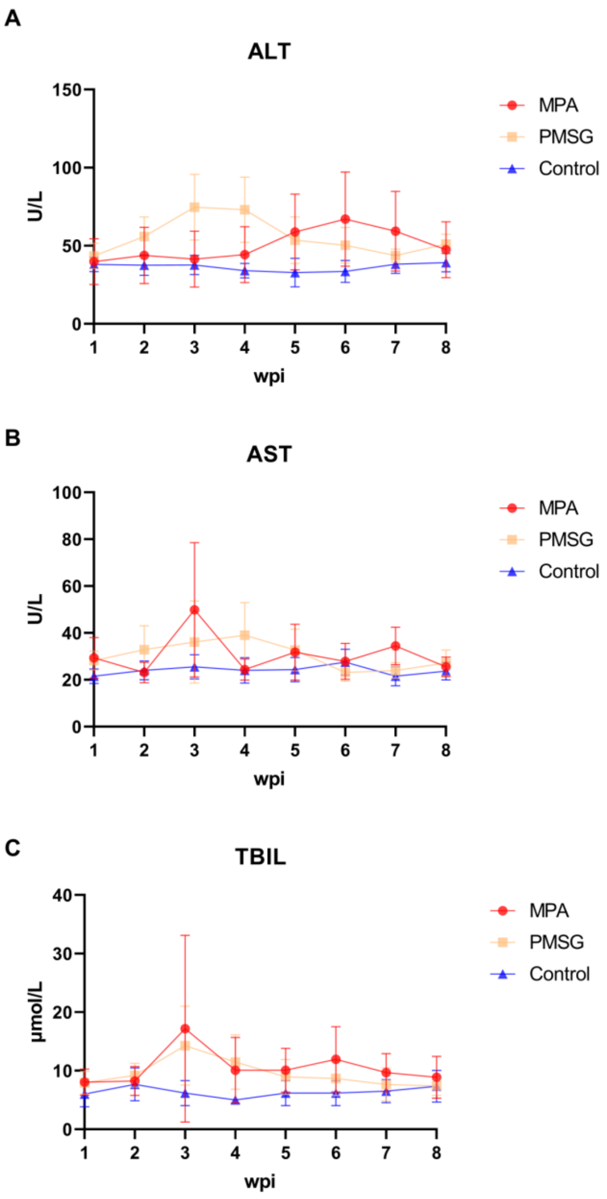

Supplementary Figure 1 Liver function of rabbits via vaginal route following medroxyprogesterone acetate (MPA), pregnant mare serum gonadotrophin (PMSG) and no hormone treatment (Control). Levels of aminotransferase (ALT) (A), aspartate aminotransferase (AST) (B) and total bilirubin (TBil) (C) in MPA, PMSG and Control groups.

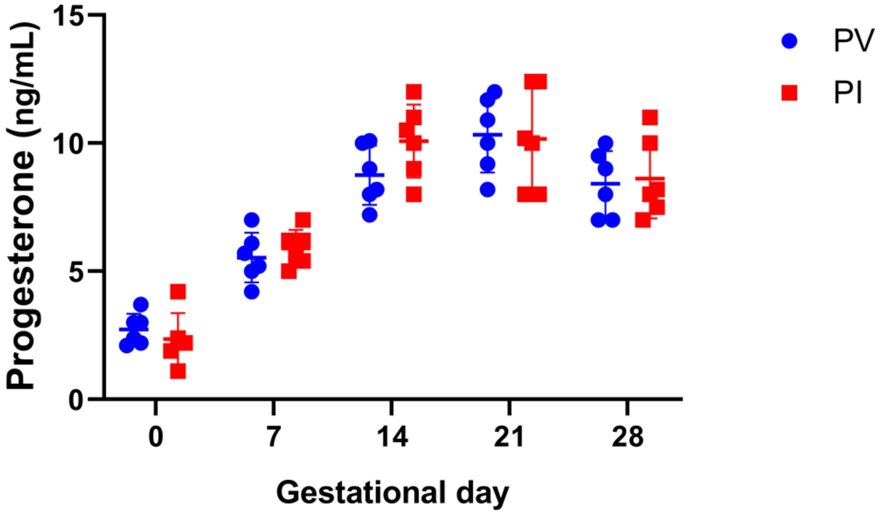

Supplementary Figure 2 The serum levels of progesterone in pregnant rabbits in groups PV and PI from gestational day 0 to 28.

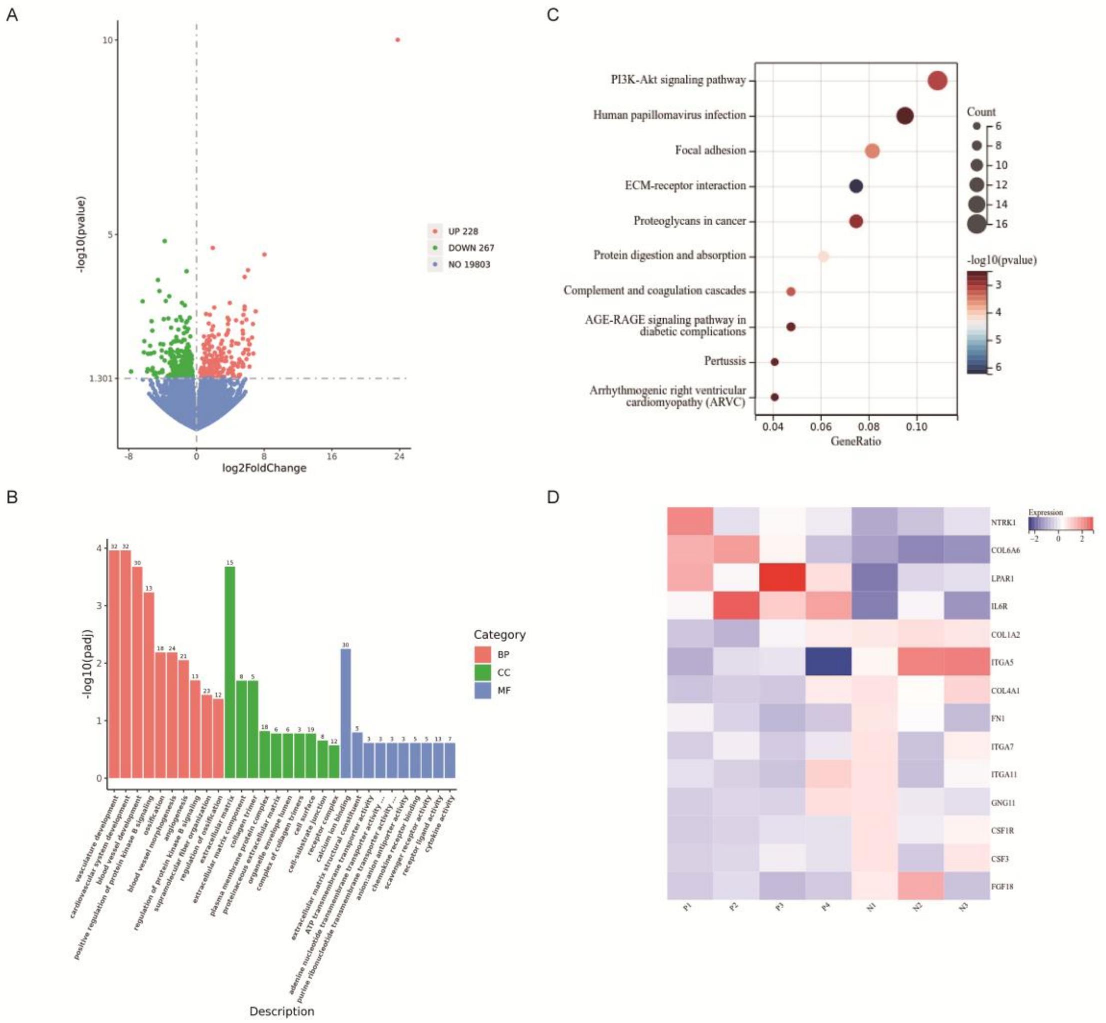

Supplementary Figure 3. Transcriptomic analysis of vaginal tissues from the Vaginal and Mock groups. (A) A total of 495 differentially expressed genes (DEGs) were identified, including 228 upregulated and 267 downregulated genes. The DEGs were identified with a fold change greater than 1 and a P-value less than 0.05. (B) Gene Ontology (GO) enrichment analysis of the identified DEGs. (C) Kyoto Encyclopedia of Genes and Genomes (KEGG) enrichment analysis of the identified DEGs.

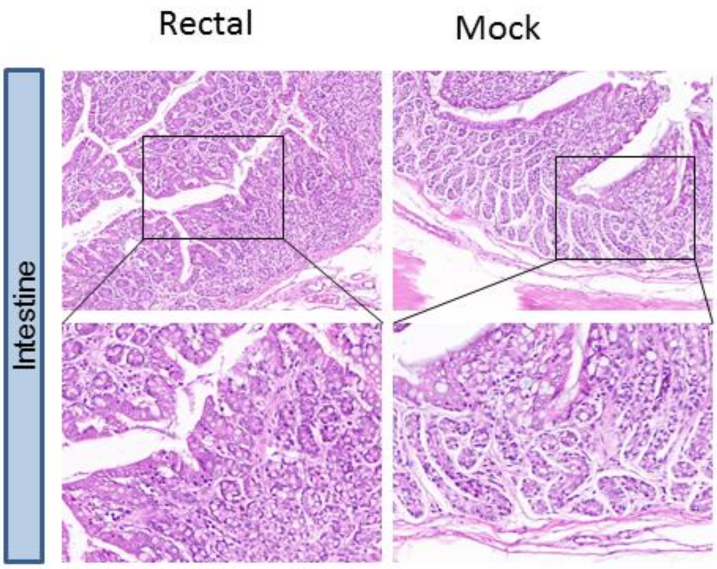

Supplementary Figure 4. Representative H&E staining of the intestine sections in Recal and Mock groups. No obvious pathological changes were observed in both groups.

Supplementary Table 1 HEV infection signs of rabbits inoculated with HEV orally

Rabbit NO.	Fecal HEV RNA				Viremia				Antigenemia				Seroconversion for anti-HEV antibodies			
	1 w pi	2 w pi	3 w pi	4 w pi	1 w pi	2 w pi	3 w pi	4 w pi	1 w pi	2 w pi	3 w pi	4 w pi	1 w pi	2 w pi	3 w pi	4 w pi
1	-	-	-	-	-	-	-	-	-	-	-	-	-	-	-	-
2	-	-	-	-	-	-	-	-	-	-	-	-	-	-	-	-
3	-	-	-	-	-	-	-	-	-	-	-	-	-	-	-	-
4	+	-	-	-	-	-	-	-	-	-	-	-	-	-	-	-
5	+	-	-	-	-	-	-	-	-	-	-	-	-	-	-	-
6	-	-	-	-	-	-	-	-	-	-	-	-	-	-	-	-
7	-	-	-	-	-	-	-	-	-	-	-	-	-	-	-	-
8	-	-	-	-	-	-	-	-	-	-	-	-	-	-	-	-
9	-	-	-	-	-	-	-	-	-	-	-	-	-	-	-	-
10	-	-	-	-	-	-	-	-	-	-	-	-	-	-	-	-
11	-	-	-	-	-	-	-	-	-	-	-	-	-	-	-	-
12	-	-	-	-	-	-	-	-	-	-	-	-	-	-	-	-
13	-	-	-	-	-	-	-	-	-	-	-	-	-	-	-	-
14	-	-	-	-	-	-	-	-	-	-	-	-	-	-	-	-
15	-	-	-	-	-	-	-	-	-	-	-	-	-	-	-	-

16	-	-	-	-	-	-	-	-	-	-	-	-	-	-	-	-
17	-	-	-	-	-	-	-	-	-	-	-	-	-	-	-	-
18	-	-	-	-	-	-	-	-	-	-	-	-	-	-	-	-
19	-	-	-	-	-	-	-	-	-	-	-	-	-	-	-	-
20	-	-	-	-	-	-	-	-	-	-	-	-	-	-	-	-

+: positive; -: negative
